# Clinical and cost-effectiveness of cardioprotection against the toxic effects of anthracyclines given to children with cancer: a systematic review

**DOI:** 10.1038/sj.bjc.6603562

**Published:** 2007-01-23

**Authors:** J Bryant, J Picot, L Baxter, G Levitt, I Sullivan, A Clegg

**Affiliations:** 1 Southampton Health Technology Assessments Centre (SHTAC), Wessex Institute for Health Research and Development, University of Southampton, Southampton SO16 7PX, UK; 2 Great Ormond Street Hospital for Children, London, UK

**Keywords:** cardioprotection, anthracyclines, children, systematic review

## Abstract

This review systematically assessed the evidence on the clinical and cost-effectiveness of cardioprotection against the toxic effects of anthracyclines given to children with cancer. We searched eight electronic databases, including Medline and the Cochrane Library, from inception to January 2006 for systematic reviews and randomised controlled trials that reported death, heart failure, arrhythmias or measures of cardiac performance associated with cardioprotective technologies compared with standard treatment in children treated for cancer with anthracyclines. Economic evaluations were also sought. Inclusion criteria, data extraction and quality assessment were undertaken by standard methodology. Four randomised controlled trials met the inclusion criteria of the review; each had methodological limitations. No economic evaluations were identified. Studies were combined through narrative synthesis. One trial found that continuous infusion of doxorubicin did not offer any cardioprotection over rapid infusion. One suggested that continuous infusion of daunorubicin provoked less cardiotoxicity than rapid infusion. One concluded that dexrazoxane reduces cardiac injury during doxorubicin therapy and one reported a protective effect of coenzyme Q_10_ on cardiac function during anthracycline therapy. The evidence on the effectiveness of cardioprotective technologies in children is limited in quality and quantity thus making conclusions difficult. This is surprising given the importance of anthracycline use in children with cancer. Further long-term research, which includes relevant outcome measures, is needed to determine whether technologies influence the development of cardiac damage without limiting the antitumour efficacy of anthracyclines.

Cytotoxic antibiotics, known as anthracyclines, are highly potent chemotherapeutic agents that have been widely used in the treatment of paediatric malignancies. Their introduction has led to the successful treatment of childhood cancer with improved survival rates now approaching 75% (Curry *et al*, 2006). There are an estimated 270 000 survivors of childhood cancer in the USA (Hewitt *et al*, 2003) and more than 20 000 in the UK (National Institute for Health and Clinical Excellence, 2005), half of whom are likely to have been treated with anthracyclines. However, as children are surviving cancer for longer, a cost of treatment, in the form of late sequelae relating to both the disease and as a consequence of therapy, has become apparent (Curry *et al*, 2006). Nearly two thirds of survivors have one or more related chronic medical problems and may require multidisciplinary care (Curry *et al*, 2006). A particular limitation of anthracycline therapy is dose-dependent cardiotoxicity that can lead to clinical heart failure (Bonadonna and Monfardini, 1969; Bu'Lock *et al*, 1996).

The problem of anthracycline-induced clinical heart failure (A-CHF) is an important public health concern as it may not be seen for many years and remains a life-long threat. It is of particular importance in children who may survive for decades after successful antineoplastic treatment. As survival rates continue to improve, the resources needed to monitor and care for survivors will also continue to increase.

The precise mechanism underlying A-CHF is not fully understood but is thought to be due, in part, to lipid peroxidation and the generation of free radicals by anthracycline–iron complexes (Myers, 1998). The heart is particularly vulnerable to free radical injury because protective enzymes are present at lower levels than in other tissues (Myers, 1998). The damage to myocardial cells may eventually lead to irreversible heart failure.

It is thought that an effective way to avoid the cardiotoxic effects of anthracyclines is to prevent cardiac injury during chemotherapy. Three approaches have been attempted: firstly by decreasing myocardial concentrations of anthracyclines and their metabolites by dose limitation or schedule modification (Lipshultz *et al*, 1998; Levitt *et al*, 2004); secondly by developing less cardiotoxic anthracycline derivatives and formulations (Batist *et al*, 2001) and thirdly by the administration of cardioprotective agents during or after chemotherapy to attenuate the effects of anthracyclines on the heart (Hellmann, 1996). The main issue of concern is that the cardioprotective technology reduces the heart damage caused by anthracyclines without reducing the antitumour efficacy and without causing other toxic effects.

Owing to the uncertainty about the effectiveness of cardioprotection and the importance of trying to reduce the impact of the A-CHF in terms of cost to the children receiving anthracyclines for cancer, their families and carers, and to the National Health Service (NHS), we were commissioned by the NHS Health Technology Assessment Programme to assess the evidence in the literature. We conducted a systematic review of cardioprotection against the toxic effects of anthracyclines given to children with cancer. This paper summarises the findings of the systematic review and discusses its implications.

## METHODS

Eight electronic databases (including Medline, Cochrane Database of Systematic Reviews, Cochrane Controlled Trials register, Embase) were searched from inception for periods up to January 2006 (search strategies available on request). Additional studies were identified through searching bibliographies of related publications and through contact with experts. Further details are available elsewhere (Bryant *et al*, in press).

Randomised controlled trials published in English language were sought that compared cardioprotective technologies with standard treatment protocols or other cardioprotective agents; included children aged up to 18 being treated for cancer with anthracyclines; and used patient-based primary outcomes of mortality, heart failure, arrhythmia or measures of cardiac performance. Systematic reviews of individual randomised trials and published economic evaluations were also sought. The quality of randomised controlled trials was assessed using criteria developed by the Centre for Reviews and Dissemination (CRD, 2001). Inclusion criteria, decisions about quality criteria and data extraction were applied independently by two reviewers, with any differences in opinion resolved through discussion. Studies of clinical effectiveness were combined through narrative synthesis with full tabulation of included studies. Meta-analysis was not appropriate owing to heterogeneity of interventions.

## RESULTS

### Quantity and quality of research

Of 994 titles and abstracts, four randomised controlled trials met the inclusion criteria (Steinhertz *et al*, 1993; Iarussi *et al*, 1994; Lipshultz *et al*, 2002, 2004) (see Table 1). Each considered a different cardioprotective intervention; two considered continuous infusion *vs* rapid (bolus) infusion, one each of doxorubicin (Lipshultz *et al*, 2002) and daunorubicin (Steinhertz *et al*, 1993), and two considered cardioprotective agents, one each of coenzyme Q10 (Iarussi *et al*, 1994) and dexrazoxane (Lipshultz *et al*, 2004). No cost-effectiveness studies considering cardioprotection in children receiving anthracycline therapy for cancer were identified.

When judged using standard criteria for assessing methodological quality, the randomised controlled trials (RCTs) were of poor quality (see Table 2). All were described as randomised but it is not known if treatment allocation was concealed and details of the methods used are mostly unknown. Eligibility criteria were specified in all cases although only partially in one trial. Details of whether outcome assessors were blinded to treatment allocation was adequate in two trials (Lipshultz *et al*, 2002, 2004); patient blinding to treatment allocation was unknown (Steinhertz *et al*, 1993; Iarussi *et al*, 1994; Lipshultz *et al*, 2002) or inadequate (Lipshultz *et al*, 2004). Results were adequately presented with point estimates and a measure of variability in two trials (Iarussi *et al*, 1994; Lipshultz *et al*, 2004) and an intention-to-treat analysis in one (Iarussi *et al*, 1994). Withdrawals and dropouts are adequately described with numbers and reasons in two trials (Iarussi *et al*, 1994; Lipshultz *et al*, 2004).

### Assessment of effectiveness

#### Continuous infusion *vs* bolus infusion of Doxorubicin (Lipshultz *et al*, 2002)

This study looked at moderate dose doxorubicin administration by continuous infusion (48 h) or by bolus infusion in children with newly diagnosed acute lymphoblastic leukaemia and was designed to detect a reduction in toxicity. After doxorubicin treatment, no significant difference was demonstrated between the two groups for any cardiac characteristic measured (see Table 3). Both groups showed abnormalities of left ventricular structure and function compared with normal reference data and baseline. For example, median left ventricular fractional shortening fell significantly by approximately two s.d. in both the continuous infusion and bolus infusion groups, but there was no statistical difference between the groups. Left ventricular contractility was depressed in both groups (for bolus patients, median *z*-score=−0.70, *P*=0.006, when compared with normal population; for continuous infusion patients, median *z*-score=−0.765, *P*=0.005, when compared with normal population). Both groups showed progressive but mild subclinical cardiotoxicity. There was no difference in event-free survival between the groups.

#### Continuous infusion *vs* bolus infusion of Daunorubicin (Steinhertz *et al*, 1993)

This pilot study compared the antileukaemic efficacy of continuous infusion of daunorubicin with bolus infusion in children with newly diagnosed acute lymphoblastic leukaemia and reported cardiac function in a subgroup of patients. Less sophisticated echocardiographic techniques were used than in the study described above (Lipshultz *et al*, 2002). A median change in left ventricular fractional shortening of −6.5 units is reported for participants who received a bolus infusion of daunorubicin and +1 unit in those receiving the continuous infusion of daunorubicin (see Table 3). It is not reported whether the change is statistically significant between the two groups.

Four of the 18 participants who received the bolus infusion of daunorubicin were identified as having an important deterioration in cardiac function (defined arbitrarily as a decrease of left ventricular fractional shortening on two consecutive evaluations to abnormal levels (less than 29%) or by 10 or more percentile units from the baseline level for that particular patient to borderline function 29% at any time during treatment or follow-up). None of those who received a continuous infusion of the drug experienced this level of fractional shortening.

Results suggested that a more rapid cytoreduction occurred when daunorubicin was delivered by continuous infusion compared with bolus infusion and the study concluded that ‘continuous infusion of daunorubicin had less cardiotoxicity with faster antileukaemic activity than bolus infusion’. However, not all results are reported separately for the different treatment groups so there are no comparable groups for statistical comparison for leukaemic cell reduction and cardiac function. We are not aware of published follow-up data regarding cardiac status for these patients.

#### Anthracycline therapy with or without coenzyme Q_10_ (Iarussi *et al*, 1994)

This small study evaluated the protective effect of coenzyme Q_10_ on anthracycline-induced cardiotoxicity in children diagnosed with acute lymphoblastic leukaemia or non-Hodgkin's lymphoma. Two main cardiac outcomes, left ventricular fractional shortening and septal wall structure and function, were reported (see Table 3).

After anthracycline treatment, percentage left ventricular fractional shortening (%LVFS) was significantly decreased in both groups of children. In the group of children receiving coenzyme Q_10_ %LVFS decreased from 40.36±4.60 at baseline to 35.82±5.02 at the end of therapy (*P*<0.05), whereas in the group that did not receive coenzyme Q_10_ %LVFS decreased from 39.89±4.37 at baseline to 33.43±3.46 at the end of therapy (*P*<0.002). The study reports that ‘mean %LVFS was significantly lower in the group that received coenzyme Q_10_ compared to the mean value in the group that did not receive coenzyme Q_10_’, but probably refers to mean reduction in %LVFS, which was lower in treated patients although no *P-*value is given for this comparison. The LVFS parameter is never reported to be below the normal value. No statistical reduction in the percentage septal wall thickening (%SWT) was reported in the group that received coenzyme Q_10_. In the group that did not receive coenzyme Q_10_ %SWT was statistically decreased from 46.10±10.1 at baseline to 27.00±18.54 at end of the therapy (*P*<0.01). No between-group statistical comparison is reported. Again, we are not aware of published follow-up data for these patients.

#### Doxorubicin therapy with or without dexrazoxane (Lipshultz *et al*, 2004)

This study was conducted to determine whether dexrazoxane therapy reduces myocardial injury as measured by serum cardiac troponin T (cTnT) levels in children with newly diagnosed acute lymphoblastic leukaemia being treated with doxorubicin (see Table 3). Echocardiographic results were available for a subgroup of patients who underwent randomisation.

Significantly, fewer patients in the group given dexrazoxane and doxorubicin compared with patients in the doxorubicin group had any elevations in cTnT (21 *vs* 50%, *P*<0.001), any extreme elevations in cTnT (10 *vs* 32%, *P*<0.001), or multiple elevations in cTnT (12 *vs* 37%, *P*<0.001).

There were no significant differences between the children who received doxorubicin alone and those who received dexrazoxane and doxorubicin in terms of mean left ventricular dimension, fractional shortening or contractility, before, during or after therapy. Fractional shortening was significantly depressed in both randomised groups during and after treatment (mean *z*-score, −1.06; *P*<0.001).

The rate of event-free survival at 2.5 years was 83% in both groups and continuous remission was 81% in both groups.

## DISCUSSION

This review, guided by an expert advisory panel, considered systematically the evidence on the clinical and cost-effectiveness of cardioprotection against the toxic effects of anthracyclines given to children with cancer. Only randomised controlled trials were included in the review as the potential for confounding is high, where children at particular risk of cardiac damage could be treated differently in terms of drug choice and dose from those at lower risk. Four randomised controlled trials were found that met the inclusion criteria of the review.

It is difficult to make conclusions about the effectiveness of technologies for reducing or preventing anthracycline-induced cardiotoxicity in children with cancer as the evidence is limited in both quantity and quality. The four RCTs considered different interventions and each had methodological limitations that may impact on results and bring into question the reliability of the conclusions. The main concern is that not all of the studies report the effect of the cardioprotective agent in terms of both subclinical and long-term cardiac damage, which may be subtle, and antitumour effectiveness through event-free survival. Two RCTs considered continuous infusion *vs* bolus infusion; one RCT found that continuous infusion of doxorubicin did not offer cardioprotection over bolus; the other suggested that continuous infusion of daunorubicin had less cardiotoxicity than bolus infusion but did not report results separately for all outcomes for the different treatment arms of the study. Two studies investigated protective agents; one concluded that dexrazoxane prevents or reduces cardiac injury as reflected in elevation of a cardiac marker (cTnT) during doxorubicin therapy for childhood acute lymphoblastic leukaemia without compromising antileukaemic efficacy of doxorubicin but it did not provide direct correlation of echocardiographic abnormality with elevations in cTnT; the other reported a protective effect of coenzyme Q_10_ on cardiac function during anthracycline therapy but did not support this conclusion with statistical data. Such inconclusive evidence on cardioprotective interventions has also been found for adults receiving anthracyclines for cancer (van Dalen *et al*, 2005).

There is no agreed consensus of the definition of cardiotoxicity and no standardisation for monitoring cardiac performance. A range of cardiac measures are reported in the included studies, which makes comparison of study results difficult. These issues need to be addressed in order to make future synthesis of evidence possible. All clinically relevant outcomes are important, including acute and chronic side effects of cardioprotective measures, because, for example, the lower peak benefit of continuous infusion may be offset by damage caused by more prolonged exposure time (Lipshultz *et al*, 2002). Overall, the most important outcome is event-free survival in terms of the whole treatment protocol. This is because if the cardioprotective agent causes increased morbidity even without impairing the anthracycline tumour killing activity, other treatments may not be given in a timely manner which may have a negative impact on final outcome.

No cost-effectiveness studies were identified, although costs are an important issue. Costs associated with anthracycline use in children with cancer relate to the cost of cardiac surveillance and the cost of management. All survivors require at least occasional echocardiography and high-risk patients require more specialist cardiological assessment, although the nature and frequency of cardiac surveillance required is controversial. Management costs include clinical supervision and drug therapy in those with abnormal cardiac function, the unproven aim being to ameliorate the progression to irreversible heart failure. In addition, there are indirect costs associated with sick long-term survivors with cardiac dysfunction who may be limited in their occupation and productivity, with reduced quality of life and social independence, which are important considerations. Technologies to reduce or prevent cardiotoxicity could influence such costs and lead to either reduced or increased costs. It may be, for example, that longer infusion time could be associated with extra costs owing to more hospitalisations and complications (Lipshultz *et al*, 2002).

Protocols used for the treatment of childhood cancer are continually developing with new drugs and new combinations of drugs proving successful, and these must balance achieving a cure and minimising side effects. Although the studies identified by this systematic review do not all reflect current UK treatment protocols, the risk of cardiotoxicity continues. Current treatment aims at intensity for high-risk patients and reduced therapy for low-risk groups. Therefore, there will continue to be some groups of patients at risk of A-CHF and subclinical toxicity may be present even in those who have had comparatively safe doses of anthracyclines. The results of the systematic review suggest that this problem has not been solved and will continue to be a major public health concern, especially in the light of increasing rates of childhood cancer (National Institute for Health and Clinical Excellence, 2005). In the meantime, anthracycline dose limitation remains the most effective tool in limiting the severity of cardiac damage in current clinical practice (Sorensen *et al*, 2003).

Further research in the form of well-designed RCTs with economic evaluations is needed. This should be conducted within the context of treatment protocols with long-term follow-up in order to determine cost-effectiveness and whether the various cardioprotective technologies influence event-free survival and cardiac outcomes in children with cancer receiving anthracyclines.

## Figures and Tables

**Table 1:** Clinical effectiveness studies

**Study, Author and date**	**Intervention and comparator**
Lipshultz *et al* (2002)	Continuous infusion of doxorubicin *vs* bolus infusion
Steinhertz *et al* (1993)	Continuous infusion of daunorubicin *vs* bolus infusion
Iarussi *et al* (1994)	Co-enzyme Q_10_ *vs* no co-enzyme Q_10_
Lipshultz *et al* (2004)	Dexraxozane *vs* no dexraxozane

**Table 2:** Quality assessment of included experimental studies

**Criteria**	**Lipshultz *et al* (2002)**	**Steinhertz ***et al*** (1993)**	**Iarussi *et al* (1994)**	**Lipshultz *et al* (2004)**
Was the assignment to the treatment groups really random?	Unknown	Unknown	Unknown	Adequate
Was the treatment allocation concealed?	Unknown	Unknown	Unknown	Unknown
Were the groups similar at baseline in terms of prognostic factors?	Reported only for the participants for whom there were outcome data.	Unknown	Reported	Reported
Were the eligibility criteria specified?	Adequate	Adequate	Partial	Adequate
Were outcome assessors blinded to the treatment allocation?	Adequate	Unknown	Unknown	Adequate
Was the care provider blinded?	Unknown	Unknown	Unknown	No
Cointerventions described	Inadequate	Adequate	Partial	Partial
Was the patient blinded?	Unknown	Unknown	Unknown	No
Were the point estimates and measure of variability presented for the primary outcome measure?	Partial	Inadequate	Adequate	Adequate
Did the analyses include an intention to treat analysis?	Inadequate	Inadequate	Adequate	Inadequate
Were withdrawals and dropouts completely described?	Inadequate	Inadequate	Adequate	Adequate

**Table 3:** Summary of effectiveness of cardioprotection from included RCTs

**Study**	**Intervention**	**Patients**	**Follow-up**	**Cardiac outcomes**	**Survival**
Lipshultz *et al* (2002)	DOX bolus (*n*=62) *vs* DOX continuous infusion (*n*=55)	ALL	0–56 months	LVFS median *z*-score after treatment, DOX bolus −0.47, DOX infusion −0.44 (*P*=0.60); LV contractility median *z*-score after treatment, DOX bolus −0.70, DOX infusion −0.765 (*P*=0.99); No congestive heart failure in either group	5-year EFS (excluding early treatment failures, mean±s.e.), DOX bolus 89.0±3.9%, DOX infusion 87.3±4.5% (*P*=0.50); 5-year EFS (including early treatment failures, mean±s.e.), DOX bolus 80±4%, DOX infusion 86±4% (*P*=0.23)
Steinhertz *et al* (1993)	DAUN bolus (*n*=22) *vs* DAUN continuous infusion (*n*=22)	ALL	Minimum 25 months	Change in LVFS, DAUN bolus −6.5 units, DAUN infusion+1 unit (*P*-value not reported); Important deterioration in cardiac function, DAUN bolus 4/18, DAUN infusion 0/18 (*P*=0.10)	With only two relapses at the time of reporting the authors were unable to see any effects on long-term survival
Iarussi *et al* (1994)	Addition of coenzyme Q_10_ (*n*=10) *vs* no coenzyme Q_10_ (*n*=10)	ALL or NHL	Not reported	%LVFS (mean±s.d.) decrease from baseline to end value, coenzyme Q_10_ 40.36±4.60–35.82±5.02, no coenzyme Q_10_ 39.89±4.37–33.43±3.46 (*P*-value not reported but difference described as significant); %SWT (mean±s.d.) decrease from baseline to end value, Coenzyme Q_10_ 44.10±13.20–40.10±15.30 (NS), no coenzyme Q_10_ 46.10±10.10–27.0±18.54 (*P*<0.01); No significant changes in left ventricular posterior wall thickening reported for either group	Not reported
Lipshultz *et al* (2004)	DOX (*n*=101) *vs* DZX and DOX (*n*=105)	ALL	Median 2.7 years	Patients with elevated cTnT levels at any time, DOX 50% (95% CI 38, 62),DZX and DOX 21% (95% CI 13, 31) (*P*<0.001); Multiple elevations, DOX 37% (95% CI 26, 49), DZX and DOX 12% (95% CI 6, 21) (*P*<0.001); Any extreme elevation, DOX 32% (95% CI 21, 43), DZX and DOX 10% (95% CI 4, 18) (*P*<0.001); Echocardiographic data, no significant differences between groups in LVFS, LVD or LVC; LVFS was significantly depressed in both groups during and after treatment (mean *z*-score −1.06, *P*<0.001)	EFS at 2.5 year, DOX 83%, DZX and DOX 83% (*P*=0.87)

ALL=acute lymphoblastic leukaemia; DAUN=daunorubicin; DOX=doxorubicin; DZX=dexrazoxane; EFS=event-free survival; LVFS=left ventricular fractional shortening; NHL=Non-Hodgkin's lymphoma; NS=not significant; SWT=septal wall thickening.
